# Nutritional and Phyto-Therapeutic Value of the Halophyte *Cladium mariscus* L. (Pohl.): A Special Focus on Seeds

**DOI:** 10.3390/plants11212910

**Published:** 2022-10-29

**Authors:** Maria João Rodrigues, Luísa Custódio, Débora Mecha, Gokhan Zengin, Zoltán Cziáky, Gyula Sotkó, Catarina Guerreiro Pereira

**Affiliations:** 1 Centre of Marine Sciences CCMAR, Faculty of Sciences and Technology, Campus of Gambelas, University of Algarve, 8005-139 Faro, Portugal; 2 Department of Biology, Science Faculty, Selcuk University, 42130 Konya, Turkey; 3 Agricultural and Molecular Research and Service Institute, University of Nyíregyháza, 4400 Nyíregyháza, Hungary; 4 Sotiva Seed Ltd., 4440 Tiszavasvári, Hungary

**Keywords:** sawgrass, seeds, nutritional value, enzyme inhibition, antioxidant, food ingredient, polyphenols

## Abstract

This work searched for the phyto-therapeutic potential and nutritional value of seeds from the halophyte *Cladium mariscus* L. (Pohl.), aiming at its use as a source of bioactive ingredients for the food industry. Hence, the nutritional profile, including minerals, of seeds biomass was determined; food-grade samples were prepared, and their phytochemical fingerprinting assessed. Extracts were evaluated for *in vitro* antioxidant potential, inhibitory capacity towards enzymes related to neuroprotection, diabetes, and hyperpigmentation, and anti-inflammatory properties, along with a toxicological assessment. Sawgrass seeds can be considered a proper nutritional source with a good supply of minerals. All extracts had a high level of total phenolics (65.3–394.4 mg GAE/g DW) and showed a chemically rich and diverse profile of metabolites that have several biological properties described (e.g., antioxidant, anti-inflammatory). Extracts had no significant toxicity (cell viabilities > 80%) and were overall strong antioxidants (particularly at radical scavenging and reducing iron), effective tyrosinase inhibitors (55–71 mg KAE/g DW), showed anti-inflammatory properties (30–60% NO decrease), and had moderate capacity to inhibit enzymes related to neuroprotection (AChE 3.7–4.2, BChE 4.3–6.0 mg GALE/g DW) and diabetes (α-glucosidase 1.0–1.1, α-amylase 0.8–1.1 mmol ACAE/g). Altogether, results suggest that sawgrass seeds have the potential to be exploited as a new food product and are a reservoir of bioactive molecules with prospective applications as ingredients for value-added, functional, and/or preservative food products.

## 1. Introduction

Plant seeds are primarily produced for reproduction and nutrition of the developing plants; however, they also significantly contribute to human nutrition, meeting most of our energy needs [1,2]. Common edible seeds include grains (wheat, corn, and rice), legumes (soybean, lentils, chickpeas, black-eyed peas), nuts (almonds, walnuts, hazelnuts, pistachios), cocoa and coffee beans, and are the base for several food products, including breads, pasta, or refined products (biscuits, pastries, and cakes) [2,3]. Seeds also provide most culinary oils and widely consumed beverages, such as coffee. Still, there are many other edible seeds, including pepper, mustard, cumin, flax, hemp, pumpkin, sesame, chia, and sunflower seeds [3]. Seeds contain mostly proteins, being an important source of calories, carbohydrate, minerals, B-vitamins, and essential amino acids [1,4]. Besides, seeds are also used for medicinal purposes all over the world due to their interesting contents in polyphenols with strong antioxidant properties, and used to manage various diseases, such as cancer, diabetes, or cardiovascular disorders [3,4,5,6].

Global environmental alterations caused by climate change are decreasing arable areas, reducing freshwater availability for agriculture, and increasing water and soil salinization, which poses a challenge to agricultural productivity and nutritional content of common food crops, limiting food production, and leading to rising prices [7,8]. This drives the need to find alternative food sources from non-conventional species that can grow in arid regions while still providing high-quality nutrients under extreme drought and salinity conditions [9]. Halophyte plants, naturally adapted to drought and high salinity, are considered by the United Nations (UN) as promising agricultural alternatives for the sustainable production of crops that use brackish and salt water for irrigation [10]. In this sense, seeds of these plants are thus promising alternatives to the traditional glycophyte seed crops. Commercial products from halophyte seeds are very scarce but there is one current widespread example, quinoa seeds (*Chenopodium quinoa* Willd.) [11]. Besides, seeds from other halophyte species are being studied mostly for extraction of edible vegetable oils, namely *Cakile maritima* Scop., *Crithmum maritimum* L., *Zygophyllum album* L.f. [12], *Portulaca oleracea* L. [13], *Arthrocnemum macrostachyum* (Moric.) Moris, *Cressa cretica* L., *Nitraria sibirica* Pall., *Salicornia* spp., and *Sueada* spp. [14].

**Figure 1:**
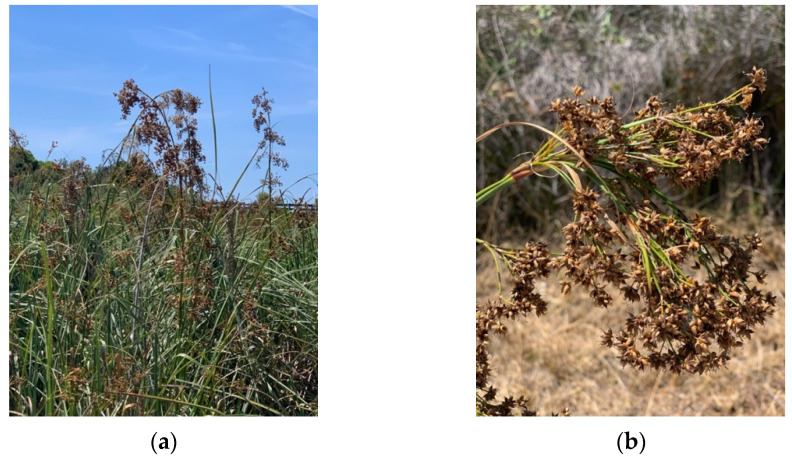
*Cladium mariscus* (sawgrass) in a saltmarsh of Southern Portugal, in the Ria Formosa Lagoon: (**a**) whole plant; (**b**) seeds. Photos by Marta Oliveira.

*Cladium mariscus* (L.) Pohl, commonly known as sawgrass (Figure 1), is a perennial evergreen halophyte that grows in saltmarshes in the Mediterranean and North Africa areas [15], and it has been used as folk medicine to treat colds, renal and gastrointestinal pain [16,17]. Sawgrass aerial parts were already described with high content in polyphenols, flavonoids and tannins, strong antioxidant, and anti-inflammatory properties. Conversely, it showed limited nutritional interest as animal feed, displaying low crude protein, high indigestible fiber contents, and very low *in vitro* digestibility [18,19]. Nevertheless, to the best of our knowledge, the phyto-therapeutic potential and nutritive value of sawgrass seeds were not previously studied. Hence, this work aimed at its valorization as a source of bioactive natural ingredients and/or novel functional food products, through the (1) determination of nutritional profile and mineral content of crude seeds biomass, (2) assessment of phytochemical composition of food grade eco-friendly seeds extracts, (3) evaluation of extracts’ *in vitro* antioxidant capacity, inhibitory potential towards enzymes related with neurodegeneration, type 2 diabetes mellitus (T2DM), and hyperpigmentation and food oxidation, and anti-inflammatory properties, and (4) determination of the extracts’ toxicological profile towards mammalian cell lines.

## 2. Results and Discussion

### 2.1. Nutritional Profile

Proper nutrition is important to prevent potentially lifestyle-related diseases such as diabetes, obesity, cardiovascular conditions, or metabolic syndrome [20]. Seeds have long been a part of the human diet because of their nutritional content [21]. In this sense, the nutritional value of sawgrass seeds was assessed, and results are presented in Table 1. The ash content (3.5 g/100 g, Table 1) in sawgrass seeds was slightly lower than that reported for its aerial parts (4.9–8.3 g/100 g) [19], but comparable to values described for edible seeds, as for example, quinoa (2.3–4.8 g/100 g) [11], cardoon (*Cynara cardunculus* L.) (2.0–3.9 g/100 g) [22], chia (*Salvia hispanica* L.) (3.5–5.0 g/100 g) [23], or sesame (*Sesamum indicum* L.) (4.5 g/100 g) [24]. Oppositely, seeds crude protein (6.5 g/100 g, Table 1) was within the range found in this halophyte’s aerial parts (5.2–8.7 g/100 g) [19], but lower than reported for quinoa (11.2–18.1 g/100 g) [11], cardoon (25.7–30.4 g/100 g) [22], chia (15.0–25.0 g/100 g) [23], and sesame (25.5 g/100 g) [24] seeds. Total lipids determined in sawgrass seeds were particularly low (0.98 g/100 g, Table 1) when compared to its aerial parts (48.9–53.3 g/100 g) [19], and also lower than that reported for quinoa (4.0–7.9 g/100 g) [11], cardoon (17.3–23.7 g/100 g) [22], chia (16.0–34.0 g/100 g) [23], and sesame (49.7 g/100 g) [24] seeds. Carbohydrates in sawgrass seeds (89.0 g/100 g), on the other hand, were slightly higher than in its aerial parts (78.8–85.0 g/100 g, estimated from reported values) [19], and also higher than that described for quinoa (48.6–68.1 g/100 g) [11], cardoon (44.0–52.2 g/100 g) [22], chia (26.0–45.0 g/100 g) [23], and sesame (24.8 g/100 g) [24] seeds. As for metabolizable energy (ME) in this halophyte, seeds (390.8 kcal/100 g) showed values similar to its aerial parts (393–405 kcal/100 g, estimated) [19]; these values are within the range of the ME determined for quinoa (275–416 kcal/100 g, estimated) [11] and chia seeds (308–586 kcal/100 g, estimated) [23], although lower than reported for cardoon (471–511 kcal/100 g) [22] and sesame (559 kcal/100 g) [24] seeds. Seeds from other halophytes, namely *Aeluropus lagopoides* (L.) Thwaites, *Eragrostis ciliaris* (L.) R.Br., *E. pilosa* (L.) P.Beauv., *Panicum antidotale* Retz., and *Sporobolus ioclados* (Nees ex Trin.) Nees, have reported values for proximate composition lower in terms of carbohydrates (32–55 g/100 g), but similar for ME (320–376 kcal/100 g) and higher in protein (10–29 g/100 g) and ash (4–9 g/100 g) [25].

Minerals are essential nutrients in the human diet, required in sufficient amounts to maintain health and normal function. Sawgrass seeds showed a high mineral content, particularly in K and Mg for macro-elements (1164 and 116 mg/100 g, respectively; Table 1), and Fe and Mn for trace-elements (3.37 and 2.57 mg/100 g, respectively; Table 1), when considering daily dietary reference values for adults (≥18 years) [26], in terms of adequate intake (AI, average nutrient level assumed as adequate for the population’s needs) or average requirement (AR, average nutrient intake that meets the daily needs of a typical healthy population). In fact, 100 g of this halophyte’s seeds (DW) can supply up to 33% of K, 39% of Mg, and 87% of Mn daily dietary adequate intakes, and up to 57% of the Fe daily average requirements (AI/day: K, 3500 mg; Mg, 300–350 mg; Mn, 3 mg; AR/day: Fe, 6–7 mg) [26]. Fe, whose deficit is the most common nutritional deficiency, is vital for energy metabolism, oxygen transport, electron transfer, and oxidase activities. Mg, being a cofactor in many ATP-involving enzymatic reactions, is essential in neuromuscular and cardiovascular systems; Mg and Mn are involved in carbohydrate, lipid, nucleic acid, and proteins metabolism. K, the most osmotically active element in cells, plays a role in cell metabolism, energy transduction, hormone secretion, and protein synthesis [26]. Additionally, sawgrass seeds can fulfil up to 16% of Ca and 14% of Cu daily average requirements, and up to 27% of Cr daily adequate intake (AR/day: Ca, 750–860 mg; Cu, 6.2–12.7 mg; AI/day: Cr, 1.3–1.6 mg) [26], while representing only 7.6% of the Na daily safe intake (2 g/day) [26]. Still noteworthy is that potentially toxic elements like Cd and Pb were not detected (below LOQs; Table 1), while Ni levels were well below reference tolerable upper intake levels (Ni: 1 mg/day) [27], which deems sawgrass seeds safe for consumption. When comparing to its aerial parts, sawgrass seeds had lower Na, Zn, and Cr contents (143–810, 1.52–2.47, and 0.17–1.3 mg/100 g, respectively) [19], but similar amounts regarding the lower values of the range reported for Ca, Fe, Mn, and Cu (160–690, 3.23–21.4, 2.0–4.9, and 0.39–0.96 mg/100 g, respectively) [19], and higher concentrations of K and Mg (230–440 and 70–80 mg/100 g, respectively) [19]. Considering the mineral composition of edible seeds from cardoon, chia, and sesame, sawgrass seeds showed higher K and Na content (K, max. 726 mg/100 g in chia; Na, max. 24 mg/100 g in cardoon) [22,23,24], but lower in the remaining elements. Reports for quinoa seeds show great variation in mineral content among varieties and locations, but mineral levels in sawgrass seeds fall within the reported range [11]. They are also within the described values for seeds from halophytic grasses (*A. lagopoides*, *E. ciliaris*, *E. pilosa*, *P. antidotale*, *S. ioclados*) [25]. Nevertheless, mineral composition in plant tissues depends on soil and plant-environment factors [28] and may therefore vary greatly. Seeds have been demonstrated as a generally good mineral dietary source [22,23,24] and *C. mariscus* seeds contribute to this finding as they would be a valuable contribution to the daily intake of some minerals, particularly K, Mg, Fe, and Mn.

Overall, the nutritional value of sawgrass seeds is akin to that of its aerial parts, with exception of total lipids [19]; in comparison to edible seeds from quinoa, cardoon, chia, and sesame, it showed lower protein and lipid content, but comparable/higher ash, carbohydrate, and minerals [11,22,23,24]. Compared to seeds from other halophytes, it had higher carbohydrates, but lower ash and proteins [25]. Additionally, sawgrass seeds represent a good supply of minerals with respect to the dietary reference values [26]. The widely consumed quinoa, chia and sesame seeds are considered important sources of nutrients in the human diet due to their nutritional contents [11,23,24]; the cardoon seeds were also assessed as a good nutrient source [22], as were the seeds from other halophytes (*A. lagopoides*, *E. ciliaris*, *E. pilosa*, *P. antidotale*, *S. ioclados*) [25]. Comparatively, sawgrass seeds may also be appraised as a proper nutritional source with great potential to be exploited as a new food product.

### 2.2. Chemical Profile

**Figure 2:**
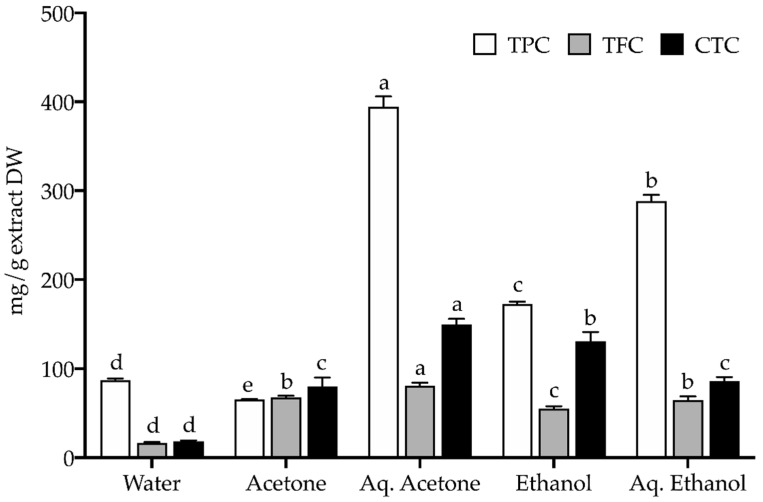
Polyphenolic contents (mg/g extract DW) in extracts of seeds from *Cladium mariscus* (sawgrass): Total phenolic content (TPC, mg GAE/g DW), Total flavonoid content (TFC, mg QE/g DW), and Condensed tannin content (CTC, mg CE/g DW). Values represent the mean ± SD (*n* = 6). For each phenolic group, different letters “a–e” represent significant differences (*p* < 0.05).

Seeds relevance in the human diet is associated not only to their nutrient content but also to polyphenolic components [21]. Ubiquitous in most plant tissues, including seeds, phenolic compounds are important secondary metabolites whose dietary intake provides potential health benefits due to their well-documented bioactive properties, such as antioxidant, anti-inflammatory, or antimicrobial, to name a few [29]. In this work, the polyphenolic contents in extracts from sawgrass seeds were assessed in terms of total phenolics (TPC), flavonoids (TFC), and condensed tannins (CTC), and results are presented in Figure 2. The aqueous acetone extract had the utmost levels of all three phenolic groups, showing it was the most effective solvent for extraction of these polyphenolic components from sawgrass seeds. The solvent type greatly influences the extraction [30] and, although there are no standardized extraction methods, water, acetone, and ethanol are ideal for the food industry since they may be used in the production of raw materials, foodstuffs, and food components/ingredients [31].

According to literature, a TPC higher than 20 mg GAE/g is indicative of natural extracts rich in phenolic compounds [18,32,33]. Hence, all sawgrass seeds extracts have a high level of phenolics since the TPC was between 65.3 and 394.4 mg GAE/g DW (acetone and aqueous acetone extracts, respectively; Figure 2). Besides having the highest TPC, the aqueous acetone extract also showed the highest flavonoid and condensed tannins contents (81.0 mg QE/g and 149.7 mg CE/g DW, respectively; Figure 2), as opposed to the water extract (16.7 mg QE/g and 18.5 mg CE/g DW, respectively; Figure 2). Other authors working with this halophyte focused on its aerial parts and report lower total phenolic (88.6 to 254 mg GAE/g) and flavonoid (13.8 to 20.2 mg QE/g), but similar tannin (38.7 to 169.6 mg CE/g) contents in their aqueous acetone extracts [18,19]. Sawgrass seeds seem comparatively richer in polyphenolic contents than their aerial organs, which may be related to different compound accumulation in different plant organs linked to the compound’s physiological role in the plant/organ interaction with their environment [34]. Still, the influence of seasonal variations on the plant’s phytochemical composition cannot be discarded. Plant material was collected in summer and environmental challenges characteristic of this season, like drought and high UV exposure, can result in enhanced levels of phenolics to cope with stressful constraints [19,35]. Compared to seeds from other halophytes (*A. lagopoides*, *E. ciliaris*, *E. pilosa*, *P. antidotale*, *S. ioclados*), sawgrass seeds displayed much higher contents in all polyphenolic groups: methanolic extracts from the former are reported to contain up to 4.2 mg GAE/g of total phenolic, 1.3 mg QE/g of total flavonoid, and 1.3 mg CE/g of tannin contents [25], which represents around 1% of the content determined in sawgrass seeds. Still noteworthy, is the sawgrass seeds high tannin content, particularly in the aqueous acetone and ethanol extracts, as tannins are recognized for their astringency, playing an important role in sensory taste perception [36], and are potentially linked to the prevention of chronic diseases such as diabetes mellitus or cardiovascular disorders [37].

**Figure 3:**
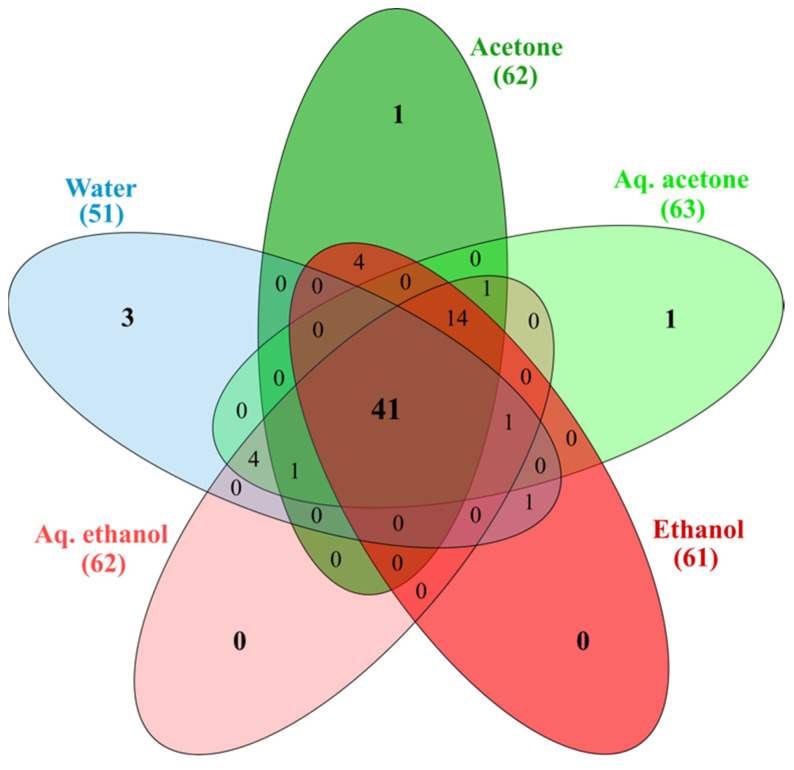
Venn diagram based on the number of identified compounds present in the different extracts from *Cladium mariscus* (sawgrass) seeds.

Sawgrass seeds’ extracts were also analyzed by HPLC-ESI-MS/MS to better understand their chemical profile, presented in Table 2 (chromatograms given in Supplementary Materials, Figures S1–S5). Identified compounds were predominantly flavonoids (30 compounds) and phenolic acids (15), along with other polyphenols (5), some fatty acids (6), stilbenes (2), and other chemicals (14). Some metabolites were identified directly, comparing retention time, exact mass, and fragment information with standards, others, with data from literature and our previous works. Results show a variation in the composition of the different extracts, depending on the extraction solvent. The water extract had the lowest number of identified molecules (51), while for the remaining samples, it was similar (61 compounds in ethanol, 62 in acetone and aqueous ethanol, and 63 in aqueous acetone). From a total of 72 metabolites identified, 41 compounds are present in all extracts while some molecules were only identified in some samples (Figure 3, Table 2).

Three compounds were specific to the water extract, namely isomers 1 and 2 of procyanidin C and caffeoylshikimic acid (compounds 8, 19, and 32 in Table 2); luteolin-C-pentoside (44) was only found in the acetone extract, and benzoic acid (18) was present only in the aqueous acetone extract. A procyanidin dimer and luteolin-C-pentoside have been reported in sawgrass aerial organs [38], but caffeoylshikimic acid and benzoic acid are currently identified in sawgrass for the first time. Acetone and ethanol extracts, i.e., non-water related extracts, had isomers 1, 2, and 3 of dimethoxy-trihydroxy(iso)flavone (61, 63, 65) and sinapyl aldehyde (38) specific to them, while ethanol and water samples were the only ones containing isoferulic acid (39). Neither isoflavones, sinapyl aldehyde, nor isoferulic acid have been reported in literature for sawgrass. The isomers 3 and 4 of procyanidin B (25, 27), lumichrome (45), and an unidentified glucoside (28) were found only in the water-related extracts; tetrahydroxyxanthose (52) was specific to acetone, aqueous acetone, and aqueous ethanol extracts. Lumichrome and tetrahydroxyxanthose are here identified in this halophyte for the first time. The ethanol extract was the only one where quercetin (55) was not found, and the acetone sample was the only one without pinellic acid (67). Additionally, 14 compounds are absent only from the water extract, namely the flavonoids gallocatechin (7), epigallocatechin (20), isoorientin (36), luteolin-*O*-hexoside (41), isoquercitrin (42), pentahydroxy(iso)flavone (50), methoxy-pentahydroxy(iso)flavone (51), methoxy-trihydroxy(iso)flavone isomers 3 and 4 (54 and 55), dimethoxy-tetrahydroxy(iso)flavone (60), the stilbenes cudranin (37) and resveratrol (43), and the fatty acids α-linolenic and 2-hydroxyhexadecanoic acids (69 and 70). As mentioned, isoflavones have not been reported elsewhere for sawgrass, nor have pinellic acid, luteolin-*O*-hexoside, isoquercitrin, cudranin, resveratrol, and α-linolenic and 2-hydroxyhexadecanoic acids. Quercetin, (epi)gallocatechins, and isoorientin are already described in sawgrass aerial organs [19,38]. From the remaining 41 molecules common to all extracts, most are, to the best of our knowledge, presently described for the first time in this halophyte, namely: the phenolic acids quinic, malic, shikimic, trihydroxybenzoic, protocatechuic, vanillic, chlorogenic, caffeic and coumaric acids; the flavonoids taxifolin, isovitexin, eriodictyol, apigenin, chrysoeriol, and isoflavones; the other polyphenols uralenneoside, vanillin, and syringaldehyde; the di/tri-carboxylic acids citric, azelaic, sebacic and undecanedioic acids; the fatty acids hydroxy-dodecenoic and -octadecadienoic, linoleic and oleic acids; and the metabolites nicotinamide, vanilloylhexose, benzofuranecarbaldehyde, scytalone, N-trans-feruloyltyramine. The remaining compounds (gallic, hydroxybenzoic, hydroxybenzaldehyde and ferulic acids, catechin, luteolin, and procyanidins) have previously been depicted in sawgrass [19,38].

From the wide diversity of compounds identified in sawgrass seeds, most stand-out due to their biological activities reported in literature. Caffeoylshikimic acid, found only in the water extract, is a primary active ingredient in oil palm phenolics (OPP), a filtrate from the aqueous waste stream of palm oil by-products. OPP has been proposed as a cardioprotective agent potentially through antioxidative and anti-inflammatory properties [39]. Benzoic acid, identified solely in the aqueous acetone extract, has antibacterial and antifungal activities, being widely used as a preservative and flavoring agent in food, pharmaceutical, and cosmetic products [40]. Discovered in both ethanol and water extracts, isoferulic acid has been associated with anti-inflammatory [41,42], cardiovascular [43], and antioxidant properties [44,45]. Present just in water-related extracts, lumichrome is a riboflavin (vitamin B_2_) derivative that influences plant growth and development [46]. Reports on lumichrome’s bioactivities include anti-platelet aggregation [47], inhibitory activity against *Staphylococcus aureus* [48], and suppression of lung cancer cell growth [49]. The flavonoid quercetin, found in acetone and water-related extracts (i.e., in all extracts except ethanol), has several bioactivities described, namely antioxidant, antimicrobial, antitumour, anti-inflammatory, cardiovascular protection, antidiabetic, among others [50]. Described in ethanol and water-related extracts (i.e., in all extracts except acetone), pinellic acid has recognized effective adjuvant activity, improving the immune response to a vaccine [51,52]; it has also demonstrated PPAR-α/γ transactivation activities [53], which could contribute to general health improvement since PPARs are directly linked to metabolism, being main regulators in energy homeostasis and metabolic function [54]. Compounds depicted in acetone-related and ethanol-related extracts (i.e., in all extracts except water) include gallo and epigallocatechins, which are some well-known tea catechins considered to be strong antioxidants, anticancer, antimicrobial, cardiovascular protective, anti-obesity, among others [55,56]; luteolin-*O*-hexoside and -6-C-glucoside (isoorientin), the later described as antioxidant, anti-neurodegenerative, and anti-diabetic [57]; resveratrol and oxyresveratrol, reported as antioxidant, anti-diabetic, antimicrobial, anti-inflammatory, neuroprotective, anti-cancer, anti-obesity, anti-melanogenic [58,59]; isoquercitrin, shown to have antioxidant, anti-inflammatory, antidiabetic, cardioprotective, and anti-cancer activities [60]; and α-linolenic and 2-hydroxyhexadecanoic acids, the first being able to improve the blood lipid profile and with cardioprotective, anti-diabetic, anti-obesity, antioxidant, anti-inflammatory, neuroprotective, and anti-cancer properties [61,62].

Present is all extracts, procyanidins, for example, display antioxidant, anti-aging, anti-diabetic, anti-inflammatory, antimicrobial, and cardio and neuroprotective effects [63]. Isoflavones, also identified in all extracts, are described as antioxidant and as an alternative treatment for several conditions, namely for cardiovascular diseases, osteoporosis, and as hormonal substitution therapy; they are also reported as chemoprotective, being connected to a lower risk of breast, uterine, and prostate cancers formation [64]. From the remaining metabolites discovered in all five extracts, flavonoids have been widely documented for their antioxidant, anti-inflammatory, antimicrobial, and anti-cancer properties [65,66]; some, like luteolin, apigenin, and taxifolin, also show anti-neurodegenerative capacity [65]. Moreover, a flavonoid-rich diet seems to relate to a decreased risk of cardiovascular diseases [65,67]. As for phenolic acids, besides their hallmark antioxidant power, they are also well known for their anti-inflammatory, anti-diabetic, antimicrobial, anti-cancer, and cardio and neuroprotective activities [68,69]. Some also have other properties described, as for example, gallic acid as anti-hyperlipidemic, protocatechuic and caffeic acids as anti-atherosclerotic, chlorogenic as anti-hypertensive, and coumaric, ferulic, and vanillic acids as anti-obesity [69]. Furthermore, phenolic acids impart organoleptic characteristics to foods, namely through color and flavor [69]. Still noteworthy is the distinctive aromatic compound from vanilla also found in all extracts, vanillin, commonly used as additive for flavor and aroma in food and cosmetic products, with antioxidant, antimicrobial, and neuroprotective activities [70,71]. In fact, phenolic compounds, mainly due to their strong antioxidant power, are considered suitable food additives and preservatives. They have been used to prevent or delay microbial growth and contamination, to inhibit lipid oxidation and consequent food deterioration, for color retention, and as flavoring agents, all of which help prolong foods shelf-life [69,72]. Additionally, it is important to mention the ubiquitous presence of relevant fatty acids in sawgrass seeds, namely oleic, linoleic, and α-linolenic (except in water extract) acids. The monounsaturated fatty acid oleic acid (ω-9 MUFA) is a major fatty acid in dietary fat, the main one in olive oil and an important component of the regarded healthy Mediterranean diet (~15% energy intake) [73]. The PUFAs (polyunsaturated fatty acids) linoleic (ω-6 PUFA) and α-linolenic (ω-3 PUFA) acids are essential fatty acids, meaning that they are required for normal growth and development but cannot be synthesized by the human body [62,74]. They must therefore be ingested, and their primary source is mainly seeds, nuts, and vegetable oils, playing key roles in physiological functions and biological processes [74,75].

Altogether, sawgrasses seeds are herein described as chemically rich and diverse in metabolites with all the above-mentioned biological properties, the most common activities between all compounds being antioxidant, anti-inflammatory, cardiovascular protection, anti-cancer, antimicrobial, anti-diabetic, and neuroprotective. This highlights these seeds potential as a reservoir of bioactive molecules with prospective applications in the food industry as ingredients for value-added, functional, and preservative food products.

### 2.3. Bioactivity Profile

Antioxidants protect cells from oxidative stress by scavenging reactive species of oxygen, nitrogen, and sulfur (ROS, RNS, RSS, respectively), countering and/or preventing oxidative damage to cellular biomolecules. These free radical and non-radical oxidants, while essential as redox signaling molecules, can be deleterious when overproduced, resulting in damage to biomolecules such as lipids, proteins, and DNA [76]. Excessive reactive species can counteract the organism’s defence systems, creating an imbalance between pro-oxidant and antioxidant species, known as oxidative stress. Oxidative stress has been implicated in the pathogenesis of conditions such as aging, inflammation, diabetes, cancer, neurodegeneration, cardiovascular diseases, among others [77,78]. Despite being controversial, it is widely accepted that antioxidants intake can enhance protection against free radicals and mitigate associated oxidative damages, potentially preventing the onset of oxidative stress-related diseases [77]. Hence, in this work, extracts from sawgrass seeds were evaluated for their antioxidant capacity in view of its potential as a new food or food ingredient.

When assessing the *in vitro* antioxidant activity of natural matrixes, the use of various assays is encouraged [79], and therefore sawgrass seeds extracts were tested by three methods targeting radical scavenging activity (RSA) and three others based on metal-related potential (Table 3). The tested samples were overall effective scavengers of DPPH•, and ABTS•+ radicals, strong at reducing iron (FRAP), and suitable at chelating copper, but the NO-scavenging and iron-chelating properties were low. The aqueous acetone and aqueous ethanol extracts were notoriously the best in RSA towards DPPH• (EC_50_ = 0.21 and 0.38 mg/mL) and ABTS•+ (EC_50_ = 0.13 and 0.22 mg/mL), and in FRAP (EC_50_ = 0.13 and 0.36 mg/mL), being at least as effective as the positive control BHA (EC_50_ = 0.60, 0.33, 0.16 mg/mL, respectively; Table 3). These values are akin to those reported in studies of aqueous acetone extracts from sawgrass aerial parts (EC_50_ between 0.23–0.30 mg/mL for DPPH•, 0.12–0.32 mg/mL for ABTS•+, and 0.18–0.27 mg/mL for FRAP) [18,19]. Seeds from other halophytes (*A. lagopoides*, *E. ciliaris*, *E. pilosa*, *P. antidotale*, *S. ioclados*; methanolic extracts) showed much lower antioxidant activity, namely in RSA against the DPPH• radical (EC_50_ 1.1–5.86 mg/mL) [25]. The CCA was also stronger in sawgrass seeds aqueous acetone and aqueous ethanol extracts (EC_50_ = 0.83 and 1.14 mg/mL) and, although not matching the positive control activity, it was higher than reported for sawgrass aerial parts (EC_50_ 2.45–6.2 mg/mL) [19]. Attending at the often-described relation between antioxidant activity and phenolic contents [80,81], the higher antioxidant potential of the aqueous acetone and aqueous ethanol extracts could be ascribed to their phenolic contents since they had the highest values of TPC (394.4 and 288.4 mg GAE/g DW, respectively; Figure 2). Similar observations have been depicted in other halophyte studies, namely with sawgrass aerial parts [19], with seeds from the halophytic grasses *A. lagopoides*, *E. ciliaris*, *E. pilosa*, *P. antidotale*, *S. ioclados* [25], *Carpobrotus edulis* (L.) N.E. Br fruits [82], *C. maritimum* leaves [83], *Limonium algarvense* Erben flowers [33], *Lythrum salicaria* L. aerial parts [18], or *Suaeda maritima* L. Dum. edible parts [84]. Moreover, extracts from sawgrasses seeds are presently portrayed as having a rich diversity of bioactive compounds, several of which with recognized antioxidant properties (e.g.,: quercetin, isoquercitrin, isoorientin, gallo and epigallocatechins, resveratrol and oxyresveratrol, procyanidins, isoflavones, flavonoids, phenolic acids). Altogether, present results corroborate previous studies demonstrating the antioxidant potential of sawgrass [18,19], while bringing forth the strong radical scavenging and copper chelating properties of its seeds.

The pharmaceutical market has been expanding in the direction of natural products due to its increasing application in several health conditions coupled with its relative low cost and importance for around 70% of the world population that has difficulty accessing costly medication [85]. Usual therapeutic tools to prevent and/or ameliorate health conditions involve the inhibition of related key enzymes, as for example, α-glucosidase and α-amylase for diabetes (T2DM), cholinesterases (AChE, BChE) for Alzheimer’s disease and neurodegeneration, or tyrosinase for hyperpigmentation disorders [86]. Hence, efforts have been focused on identifying effective enzymatic inhibitors from natural sources [82,87,88,89,90]. In this sense, the enzyme inhibitory properties of extracts from sawgrass seeds were evaluated on the above-mentioned enzymes implicated with neurodegeneration (AChE and BChE), T2DM (α-glucosidase and α-amylase), and hyperpigmentation (tyrosinase), in view of their potential as source of phyto-therapeutic compounds and/or functional food ingredients (Table 4). To the best of our knowledge, this is the first report of sawgrass inhibitory activity towards these enzymes.

Extracts showed low activity towards α-glucosidase (1.05–1.10 mmol ACAE/g) and α-amylase (0.81–1.12 mmol ACAE/g), despite all samples having displayed inhibition (Table 4). Inhibition of these hydrolyzing enzymes decreases the rate of carbohydrate breakdown, delaying carbohydrate digestion and overall glucose absorption, which results in lowered postprandial blood glucose levels. This therapeutic approach overall reduces hyperglycaemia linked to T2DM, aiding in its management [91,92]. Thus, further exploration of sawgrass seeds hypoglycaemic properties could prove relevant considering the potential of food ingredients to help control diabetes [91]. Regarding the inhibition of cholinesterases (Table 4), extracts were moderately active towards AChE (3.73–4.21 mg GALAE/g) and BChE (3.47–6.02 mg GALAE/g), and only the ethanol sample did not show any activity on BChE. Cholinesterase inhibitors are the main therapeutic strategy to manage symptoms of the neurodegenerative Alzheimer’s disease by helping maintain normal levels of the neurotransmitter acetylcholine, inhibiting its main hydrolyzing enzyme, AChE [93,94]. They are also reported as prospectively improving cognitive function in non-Alzheimer’s dementias [95], and as potential add-on therapy in conditions such as schizophrenia [96]. Several phytochemicals with cholinesterase inhibitory activity have already been isolated, like galantamine, ursolic acid, and haloxysterols, to name a few [94], and current results suggest that sawgrass seeds also possess molecules with anti-cholinesterase activity.

Moreover, the extracts proved to be very effective tyrosinase inhibitors, particularly the aqueous acetone and aqueous ethanol samples (70.26 and 70.99 mg KAE/g, respectively; Table 4), displaying more than double the activity of extracts considered as strong inhibitors such as those from *C. edulis* fruits (22.21–29.55 mg KAE/g) [82], *C. maritima* aerial organs (19.9–25.9 mg KAE/g) [97], or goji berries *Lycium barbarum* (31.5 mg KAE/g) [98]. Tyrosinase is a multifunctional copper-containing enzyme essential in melanin biosynthesis, as it is the first enzyme in the conversion of tyrosine to melanin. Increased biosynthesis and accumulation of melanin results in melanogenic or skin hyperpigmentation disorders [99,100]. Tyrosinase is also responsible for the post-harvest enzymatic browning of fruits and vegetables that produces quinones by oxidation of phenolic compounds, which has consequent nutritional (reduction of proteins/amino acid digestibility and availability) and economical (decrease of market value) losses [101,102]. In this context, compounds able to inhibit tyrosinase activity are essential to prevent/treat pigmentation conditions and enzymatic browning of plant-derived foods [99,101]. Some of the above-described constituents of sawgrass seeds extracts (Table 2) are reported as effective natural tyrosinase inhibitors, as for example, gallic acid, quercetin, taxifolin, vanillin, azelaic acid, oxyresveratrol, catechins, and procyanidins [99,101,102], and may account for the observed activity. Overall, the presently seen strong tyrosinase inhibition of sawgrass seeds attest to its potential applications in the cosmetic and food industries as a source of ingredients with anti-hyperpigmentation properties and as a food preservative.

To complete the bioactivities profile of sawgrasses seeds, extracts were tested for anti-inflammatory properties by simulating chronic inflammation in RAW 264.7 macrophages (stimulated with bacterial lipopolysaccharide (LPS) to produce nitric oxide (NO)) and determining their ability to reduce NO production (Table 5). The inflammatory process is involved in many health problems across humans’ life span, particularly chronic inflammatory states (stroke, ischemic heart disease, cancer, neurodegenerative conditions, to name a few), and inflammation-related disorders account for more than half of worldwide mortality [103].

NO is a versatile intercellular signaling molecule in several biological processes, namely in inflammation and immune response [104]. Many immune-system cells, including macrophages, produce and respond to NO; however, NO can be a pro-inflammatory mediator whose over-production induces inflammation [104,105]. In this context, the capacity to reduce NO production can be used as proxy for extracts anti-inflammatory activity [19,106,107,108,109,110]. In this work, the aqueous ethanol extract (at 100 μg/mL) was able to reduce NO production by 60% while the remaining extracts showed a more modest NO reduction (27–38%), except for the aqueous acetone sample that had no activity (Table 5). Among the compounds currently identified in sawgrass seeds extracts (Table 2), ferulic and isoferulic acids [41], resveratrol [58], oxyresveratrol [59], isoquercitrin [60], quercetin and catechin that act synergistically [111], linolenic acid [62], procyanidins [63], flavonoids [66], and phenolic acids [68] are reported in literature as anti-inflammatory compounds. However, none of the identified compounds were exclusive of the aqueous ethanol to account for its higher capacity to reduce NO production. Perhaps higher quantities of the combination of some of those compounds in this extract can be contributing to its greater NO inhibitory capacity. Sawgrass aerial parts have previously shown anti-inflammatory capacity by reducing NO production by 30%, although in aqueous acetone extracts (at 100 μg/mL), which presently demonstrated nil activity [19]. This may be due to the presence of molecules with anti-inflammatory effects in the extract from aerial parts that were not identified in its seeds, such as syringic acid or luteolin-7-*O*-glucoside [19]. Generally, these results support the previous findings of anti-inflammatory potential for sawgrass [19], though suggesting a stronger activity in its seeds.

### 2.4. Toxicological Profile

**Figure 4:**
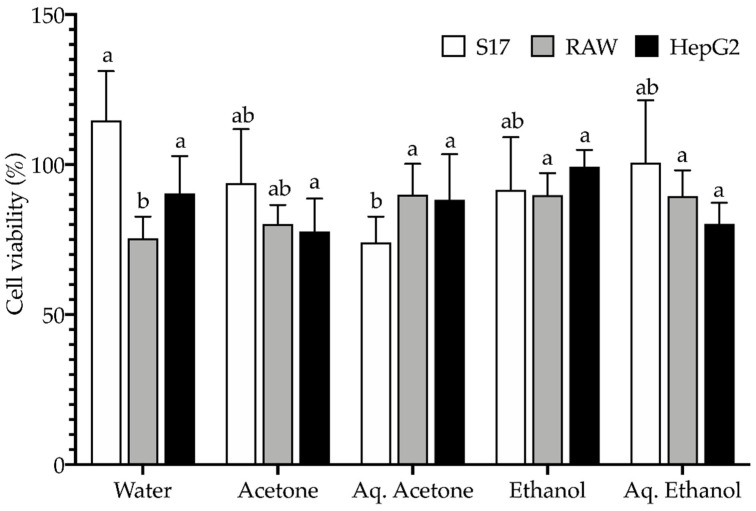
Cellular viability (%) after application of extracts (100 μg/mL extract DW) from *Cladium mariscus* (sawgrass) seeds on mammalian cell lines: S17, RAW 264.7, and HepG2. Cells treated only with cell culture medium were used as controls. Values represent the mean ± standard deviation (SD) of at least three experiments performed in triplicate (*n* = 9). For each cell line, different letters “a, b” mean significant differences (*p* < 0.05).

Natural products and ingredients are usually regarded as safer to the consumer, but it is of utmost importance to ensure their toxicological safety. This can be achieved *in vitro* by means of cellular models, which can be a preliminary proxy for toxicity assessment of natural extracts [82,97,107,112,113,114]. Hence, the extracts potential toxicity was assessed by determining cellular viability after application of the extracts on three mammalian cell lines, namely murine bone marrow stromal (S17), murine leukemic macrophage (RAW 264.7), and human hepatocarcinoma (HepG2) cells (Figure 4).

Incubation of cells with extracts at 100 μg/mL for 72 h yielded cellular viabilities almost always higher than 80% (Figure 4), therefore showing no significant toxicity. The non-tumoral cell line S17 even displayed more than 90% cellular viabilities (except for the aqueous acetone sample), with the water extract triggering an increase in S17 cell viability (~115%). No reports were found in literature for (cito)toxicity of extracts from sawgrass extracts, but other studies dealing with biotechnological applications of halophytes for consumer use account for a generally safe toxicological profile of its extracts: fruits from *C. edulis* [82], fruits and aerial organs from *C. maritima* [97], roots and aerial organs from *Artemisia campestris* subsp. *maritima* [113], stems, leaves, and flowers from *C. maritimum* [114], flowers from *L. algarvense* [107], to name a few. According to these results, sawgrass seeds extracts follow this tendency and may be considered safe to use as food components/ingredients.

## 3. Materials and Methods

### 3.1. Plant Material and Extraction

Sawgrass aerial parts (voucher code XBH03, XtremeBio lab. herbarium, Faro, Portugal) were harvested in Southern Portugal, in Ria Formosa Lagoon near Faro (37°01′03.3″ N, 7°59′18.1″ W) in Summer 2017 (July) (Figure 1). Seeds were collected from the mature inflorescences, oven-dried for 48 h at 40 °C, manually separated from the hard coat (outer envelope/seed cover), ground to a fine powder, and stored at −20 °C for further analysis. Dried powdered biomass was extracted (1:40, *w*/*v*) with water, acetone, aqueous acetone (80%), ethanol, and aqueous ethanol (80%), under stirring for 24 h, at room temperature. Extracts were filtered (Whatman filter paper grade 4) and solvents evaporated under reduced pressure at 40 °C in a rotary evaporator (R-210, Buchi Labortechnik AG, Flawil, Switzerland). Dried extracts were dissolved in DMSO (dimethyl sulfoxide) at 25 mg/mL and stored at −20 °C.

### 3.2. Nutritional Profile

Samples of dried seeds were analyzed for: ash content by incineration at 600 °C for 2 h in a muffle furnace [115]; crude protein content by measuring total nitrogen (CHN Elemental Analyzer Vario EL III) and estimating (N × 6.25) according to the macro-Kjeldahl method [116]; and crude fat (total lipids) according to a modified protocol of the Bligh and Dyer method [117]. Total carbohydrates were estimated by difference. Results are expressed as g/100 g of dried weight biomass (DW). Metabolizable energy (ME) was calculated using FAO [118] recommendations for food energy conversion factors based on the analytical methods used, according to the following equation: ME = 4 × (proteins) + 4 × (carbohydrates) + 9 × (lipids), and results are expressed as kcal/100 g DW. Dried seeds were also analyzed for minerals by Microwave Plasma-Atomic Emission Spectrometer (MP-AES; Agilent 4200 MP-AES, Agilent, Victoria, Australia), as described in Pereira et al. [114]. Prior to analysis, samples were digested with 67% nitric acid on a Microwave Digestion System (Discover SP-D 80, CEM Corp., Matthews, NC, USA) for 4 min ramp temperature to 200 °C and hold for 3 min, and diluted (1:10) with ultra-pure water. Results are expressed as mg/100 g DW.

### 3.3. Chemical Profile

#### 3.3.1. Total Phenolic, Flavonoid, and Condensed Tannin Content

The extracts’ content in total phenolics (TPC), flavonoids (TFC), and condensed tannins (CTC) was estimated by colorimetric assays adapted to 96-well microplates, namely: Folin-Ciocalteu, aluminum chloride (AlCl_3_), and 4-dimethylaminocinnamaldehyde (DMACA), respectively, as described in Oliveira et al. [19]. Gallic acid, quercetin, and catechin were the standards used in calibration curves to calculate TPC, TFC, and CTC, and results are expressed as milligrams of standard equivalents (correspondingly, GAE, QE, and CE) per gram of extract dry weight (DW).

#### 3.3.2. Phytochemical Composition by HPLC-ESI-MS/MS (High Performance Liquid Chromatography Coupled with Electrospray Ionization Mass Spectrometry)

The metabolite profiling of the extracts was assessed with a chromatographic system Dionex Ultimate 3000RS UHPLC equipped with Thermo Accucore C18 column (100 mm × 2.1 mm i.d., 2.6 µm) at 25 °C (±1 °C), as detailed in Castañeda-Loaiza et al. [82]. Prior to HPLC analysis, extracts were filtered with 0.22 µm PTFE filter membrane (Labex Ltd., Budapest, Hungary). The mobile phase was water (A) and methanol (B), both acidified with 0.1% formic acid, following a gradient elution of: (0–3 min) 5% B, (3–43 min) linear gradient from 5% to 100% B, (43–61 min) 100% B, (61–62 min) linear gradient from 100% to 5% B, and (62–70 min) 5% B (0.2 mL/min flow rate). For analysis, a Thermo Q Exactive Orbitrap MS (Thermo Fisher Scientific, Massachusetts, NC, USA) with electrospray ionization probe interface was used, in positive and negative-ion mode. Full scan was carried out upon conditions described in Zengin et al. [88]. Thermo Scientific Xcalibur 3.1 was employed for control and data processing and Trace Finder 3.1 was used for target screening (Thermo Fisher Scientific, Massachusetts, NC, USA). All spectral data of individual compounds was analyzed by exact mass, retention time, isotopic pattern, and characteristic fragment profile. Some compounds were identified by direct comparison with standards (marked as such in Table 2), while others were identified based on our previous works and/or data in literature.

### 3.4. Bioactivity Profile

#### 3.4.1. *In Vitro* Antioxidant Activity

Extracts were assessed for antioxidant capacity by three radical-based assays and three metal-related assays. Their radical scavenging activity (RSA) was evaluated towards the DPPH• (1,1-diphenyl-2picrylhydrazyl), ABTS•+ (2,2′-azino-bis(3-ethylbenzothiazoline-6-sulfonic acid)), and NO (nitric oxide) radicals using BHA (butylated hydroxyanisole) and ascorbic acid as positive controls. Their Fe^3+^ reducing capacity (ferric reducing antioxidant power, FRAP) and ability to chelate copper (CCA) and iron (ICA) were evaluated using BHA and EDTA (ethylenediaminetetraacetic acid) as positive controls. Methods are fully described in Oliveira et al. [19] and Rodrigues et al. [33]. Results were estimated as percentage of activity in relation to a negative control (DMSO), except for FRAP which was relative to the positive control, and are expressed as half maximal effective concentration, namely EC_50_ values (mg/mL).

#### 3.4.2. *In Vitro* Enzyme Inhibitory Activity

Extracts were tested for inhibitory effects towards selected enzymes, namely cholinesterases (acetyl and butyrylcholinesterase, AChE and BChE, respectively), tyrosinase, α-glucosidase, and α-amylase. Cholinesterases inhibition was evaluated by the Ellman’s method using galantamine as standard; tyrosinase inhibition was assessed by the modified dopachrome method with kojic acid as standard; for α-glucosidase and α-amylase inhibition, acarbose was the standard inhibitor. Methods are thoroughly described in Uysal et al. [87]. Results are expressed as milligrams of standard inhibitor equivalents (galantamine—GALAE, kojic acid—KAE, and acarbose—ACAE) per gram of extract.

#### 3.4.3. Cell Culture

The human hepatocarcinoma (HepG2) and murine bone marrow stromal (S17) cell lines were kindly provided by the Center for Biomedical Research (CBMR, University of Algarve, Portugal); the murine leukemic macrophage cell line (RAW 264.7) was kindly provided by the Mountain Research Center (CIMO, Bragança Polytechnic Institute, Portugal). HepG2 and S17 cells were cultured in DMEM and RAW cells in RPMI medium, both media supplemented with 10% heat-inactivated foetal bovine serum, 1% L-glutamine (2 mM), and 1% penicillin (50 U/mL)/streptomycin (50 μg/mL). Cells were kept at 37 °C under a humidified atmosphere of 5% CO_2_.

#### 3.4.4. *In Vitro* Anti-Inflammatory Activity

Extracts were evaluated for anti-inflammatory properties by stimulating RAW 264.7 macrophages with lipopolysaccharide (LPS) to produce nitric oxide (NO), as described in Rodrigues et al. [106]. Extracts were firstly assessed for cellular viability by the MTT (3-(4,5-dimethylthiazol-2-yl)-2,5-diphenyltetrazolium bromide) assay, having been incubated for 24 h with cells seeded at 10 × 10^3^ cells/well. Extracts at non-cytotoxic concentrations (allowing more than 80% cell viability) were incubated for 24 h with cells seeded at 2.5 × 10^5^ cells/well in serum and phenol-free medium, with LPS at 25 μg/mL. NO content was determined following the Griess method, using a calibration curve prepared with sodium nitrite as standard. Results are expressed as % of NO decrease relative to a control.

### 3.5. Toxicological Profile

The extracts toxicity was determined using three mammalian cell lines, namely HepG2, S17, and RAW 264.7 cells. Toxicity was assessed according to Rodrigues et al. [107], as follows. Growing cells were seeded in 96-well microplates at an initial density of 5 × 10^3^ cells/well for HepG2 and S17, and at 10 × 10^3^ cells/well for RAW, and left incubating for 24 h to allow cell adhesion. Extracts at 100 μg/mL concentration were incubated with cells for 72 h using culture medium as negative control. Cellular viability was determined by the MTT assay and results are expressed as % cellular viability.

### 3.6. Statistical Analysis

Experiments were performed in triplicate and results are expressed as the mean ± standard deviation (SD). EC_50_ values were obtained by curve fitting with GraphPad Prism 8.4.3 for Mac (GraphPad Software, Sand Diego, CA, USA). Statistical differences (*p* < 0.05) were determined by one-way ANOVA and the pairwise Tukey multiple comparison test; in the absence of data parametricity, Kruskal Wallis and Dunn’s test were used. Statistical tests were performed using XLSTAT trial version for Mac (Addinsoft 2022, New York, NY, USA).

## 4. Conclusions

This study is the first to account for a comprehensive phyto-therapeutic and nutritional assessment of *C. mariscus* seeds as a source of bioactive natural ingredients or functional food products. Results indicated that sawgrass seeds may be appraised as a suitable nutritional source with a good supply of minerals and therefore with great potential as a new food product. Its extracts showed no toxicity, are chemically rich and diverse in metabolites, and an unexplored source of strong antioxidants, effective tyrosinase inhibitors, and anti-inflammatory molecules, not discarding minor neuroprotective and anti-diabetic properties. Therefore, these seeds have great potential to be exploited as a new food product, having prospective applications as value-added, functional, or preservative food ingredients. Additionally, they could also deliver raw material to the pharmaceutical and cosmetic industry segments, to prevent/manage oxidative-stress related and skin-hyperpigmentation conditions.

## Figures and Tables

**Table 1:** Nutritional value of seeds from *Cladium mariscus* (sawgrass): proximate composition (ash, crude protein, total lipids, carbohydrates, and metabolizable energy—ME) and mineral content (macro and trace elements).

Nutritional Profile	Contents
**Proximate composition**	(g/100 g DW)
Ash	3.52 ± 0.50
Crude protein	6.55 ± 0.46
Total lipids	0.98 ± 0.07
Carbohydrates	88.96 ± 0.39
ME (kcal/100 g DW)	390.81 ± 1.46
**Mineral content**	(mg/100 g DW)
Macro elements	
Calcium (Ca)	138.57 ± 3.51
Potassium (K)	1164.31 ± 43.8
Magnesium (Mg)	116.52 ± 3.06
Sodium (Na)	152.24 ± 6.89
Trace elements	
Iron (Fe)	3.37 ± 0.29
Manganese (Mn)	2.57 ± 0.12
Zinc (Zn)	0.86 ± 0.06
Copper (Cu)	0.35 ± 0.03
Chromium (Cr)	0.07 ± 0.00
Nickel (Ni)	0.07 ± 0.00
Cadmium (Cd)	<LOQ ^1^
Lead (Pb)	<LOQ ^2^

Values represent mean ± standard deviation (SD) (*n* = 3). DW: dry weigh; LOQ: limit of quantification. LOQs: ^1^ Cd = 0.02 mg/100 g DW, ^2^ Pb = 0.05 mg/100 g DW.

**Table 2:** Metabolite profiling of extracts from *Cladium mariscus* (sawgrass) seeds by HPLC-ESI-MS/MS.

Nº	Name	Formula	RT	[M + H]^+^	[M − H]^−^	Water	Acetone	Aq. Acetone	Ethanol	Aq. Ethanol
1 ^1^	Quinic acid	C_7_H_12_O_6_	2.00		191.05557	+	+	+	+	+
2	Malic acid	C_4_H_6_O_5_	2.17		133.01370	+	+	+	+	+
3 ^1^	Shikimic acid	C_7_H_10_O_5_	2.29		173.04500	+	+	+	+	+
4	Nicotinamide	C_6_H_6_N_2_O	2.44	123.05584		+	+	+	+	+
5	Citric acid	C_6_H_8_O_7_	2.57		191.01918	+	+	+	+	+
6 ^1^	Gallic acid (3,4,5-Trihydroxybenzoic acid)	C_7_H_6_O_5_	3.33		169.01370	+	+	+	+	+
7	Gallocatechin	C_15_H_14_O_7_	5.74		305.06613	-	+	+	+	+
8	Procyanidin C isomer 1	C_45_H_38_O_18_	4.11		865.19799	+	-	-	-	-
9	Trihydroxybenzoic acid	C_7_H_6_O_5_	5.84		169.01370	+	+	+	+	+
10	Protocatechuic acid (3,4-Dihydroxybenzoic acid)	C_7_H_6_O_4_	5.96		153.01879	+	+	+	+	+
11	Vanilloylhexose	C_14_H_18_O_9_	7.81		329.08726	+	+	+	+	+
12	Hydroxybenzoic acid isomer 1	C_7_H_6_O_3_	9.37		137.02387	+	+	+	+	+
13	Hydroxybenzoic acid isomer 2	C_7_H_6_O_3_	10.42		137.02387	+	+	+	+	+
14	Procyanidin B isomer 1	C_30_H_26_O_12_	11.19		577.13460	+	+	+	+	+
15	Uralenneoside	C_12_H_14_O_8_	11.24		285.06105	+	+	+	+	+
16	Procyanidin B isomer 2	C_30_H_26_O_12_	12.41		577.13460	+	+	+	+	+
17	Hydroxybenzaldehyde	C_7_H_6_O_2_	12.96	123.04461		+	+	+	+	+
18	Benzoic acid	C_7_H_6_O_2_	12.97		121.02896	-	-	+	-	-
19	Procyanidin C isomer 2	C_45_H_38_O_18_	13.31		865.19799	+	-	-	-	-
20 ^1^	Epigallocatechin	C_15_H_14_O_7_	13.52		305.06613	-	+	+	+	+
21 ^1^	Catechin	C_15_H_14_O_6_	13.57		289.07121	+	+	+	+	+
22	Vanillic acid (4-Hydroxy-3-methoxybenzoic acid)	C_8_H_8_O_4_	14.06		167.03445	+	+	+	+	+
23 ^1^	Chlorogenic acid (3-O-Caffeoylquinic acid)	C_16_H_18_O_9_	14.38	355.10291		+	+	+	+	+
24	Caffeic acid	C_9_H_8_O_4_	14.63		179.03444	+	+	+	+	+
25	Procyanidin B isomer 3	C_30_H_26_O_12_	15.21		577.13460	+	-	+	-	+
26 ^1^	Vanillin (4-Hydroxy-3-methoxybenzaldehyde)	C_8_H_8_O_3_	15.91	153.05517		+	+	+	+	+
27	Procyanidin B isomer 4	C_30_H_26_O_12_	16.92		577.13460	+	-	+	-	+
28	Unidentified glucoside	C_14_H_24_O_10_	17.06		351.12912	+	-	+	-	+
29	Syringaldehyde (3,5-Dimethoxy-4-hydroxybenzaldehyde)	C_9_H_10_O_4_	17.45	183.06574		+	+	+	+	+
30	1-Benzofuranecarbaldehyde	C_9_H_6_O_2_	17.90	147.04461		+	+	+	+	+
31 ^1^	4-Coumaric acid	C_9_H_8_O_3_	17.94		163.03952	+	+	+	+	+
32	Caffeoylshikimic acid	C_16_H_16_O_8_	17.97		335.07670	+	-	-	-	-
33 ^1^	Taxifolin (Dihydroquercetin)	C_15_H_12_O_7_	19.25		303.05048	+	+	+	+	+
34	Scytalone or isomer	C_10_H_10_O_4_	19.32	195.06574		+	+	+	+	+
35 ^1^	Ferulic acid	C_10_H_10_O_4_	19.34		193.05009	+	+	+	+	+
36	Isoorientin (Luteolin-6-C-glucoside)	C_21_H_20_O_11_	20.36	449.10839		-	+	+	+	+
37	Cudranin (Oxyresveratrol)	C_14_H_12_O_4_	20.38	245.08139		-	+	+	+	+
38	Sinapyl aldehyde (3,5-Dimethoxy-4-hydroxycinnamaldehyde)	C_11_H_12_O_4_	20.42	209.08139		-	+	-	+	-
39	Isoferulic acid	C_10_H_10_O_4_	20.51		193.05009	+	-	-	+	-
40	Isovitexin (Apigenin-6-C-glucoside)	C_21_H_20_O_10_	21.98	433.11348		+	+	+	+	+
41	Luteolin-O-hexoside	C_21_H_20_O_11_	22.06		447.09274	-	+	+	+	+
42 ^1^	Isoquercitrin (Quercetin-3-O-glucoside)	C_21_H_20_O_12_	22.56		463.08765	-	+	+	+	+
43 ^1^	Resveratrol	C_14_H_12_O_3_	22.83	229.08647		-	+	+	+	+
44	Luteolin-C-pentoside	C_20_H_18_O_10_	23.13	419.09783		-	+	-	-	-
45	Lumichrome	C_12_H_10_N_4_O_2_	23.80	243.08821		+	-	+	-	+
46	Methoxy-trihydroxy(iso)flavone isomer 1	C_16_H_12_O_6_	24.34		299.05556	+	+	+	+	+
47	N-trans-Feruloyltyramine	C_18_H_19_NO_4_	24.53	314.13924		+	+	+	+	+
48	Azelaic acid (Nonanedioic acid)	C_9_H_16_O_4_	24.63		187.09704	+	+	+	+	+
49 ^1^	Eriodictyol (3′,4′,5,7-Tetrahydroxyflavanone)	C_15_H_12_O_6_	24.89		287.05556	+	+	+	+	+
50	Pentahydroxy(iso)flavone	C_15_H_10_O_7_	25.40	303.05048		-	+	+	+	+
51	Methoxy-pentahydroxy(iso)flavone	C_16_H_12_O_8_	25.56		331.04540	-	+	+	+	+
52	Tetrahydroxyxanthone	C_13_H_8_O_6_	25.84		259.02427	-	+	+	-	+
53	Methoxy-trihydroxy(iso)flavone isomer 2	C_16_H_12_O_6_	26.01		299.05556	+	+	+	+	+
54	Methoxy-trihydroxy(iso)flavone isomer 3	C_16_H_12_O_6_	26.38		299.05556	-	+	+	+	+
55 ^1^	Quercetin (3,3′,4′,5,7-Pentahydroxyflavone)	C_15_H_10_O_7_	26.72		301.03483	+	+	+	-	+
56 ^1^	Luteolin (3′,4′,5,7-Tetrahydroxyflavone)	C_15_H_10_O_6_	27.55		285.03991	+	+	+	+	+
57	Methoxy-trihydroxy(iso)flavone isomer 4	C_16_H_12_O_6_	27.70		299.05556	-	+	+	+	+
58	Sebacic acid (Decanedioic acid)	C_10_H_18_O_4_	28.11		201.11268	+	+	+	+	+
59 ^1^	Apigenin (4′,5,7-Trihydroxyflavone)	C_15_H_10_O_5_	29.44		269.04500	+	+	+	+	+
60	Dimethoxy-tetrahydroxy(iso)flavone	C_17_H_14_O_8_	29.51		345.06105	-	+	+	+	+
61	Dimethoxy-trihydroxy(iso)flavone isomer 1	C_17_H_14_O_7_	29.57		329.06613	-	+	-	+	-
62	Chrysoeriol (3′-Methoxy-4′,5,7-trihydroxyflavone)	C_16_H_12_O_6_	29.63		299.05556	+	+	+	+	+
63	Dimethoxy-trihydroxy(iso)flavone isomer 2	C_17_H_14_O_7_	30.45		329.06613	-	+	-	+	-
64	Undecanedioic acid	C_11_H_20_O_4_	31.02		215.12834	+	+	+	+	+
65	Dimethoxy-trihydroxy(iso)flavone isomer 3	C_17_H_14_O_7_	31.36		329.06613	-	+	-	+	-
66	Hydroxydodecenoic acid	C_12_H_22_O_3_	32.47		213.14907	+	+	+	+	+
67	Pinellic acid	C_18_H_34_O_5_	33.61		329.23280	+	-	+	+	+
68	Hydroxyoctadecadienoic acid	C_18_H_32_O_3_	41.09		295.22732	+	+	+	+	+
69 ^1^	α-Linolenic acid	C_18_H_30_O_2_	44.82		277.21676	-	+	+	+	+
70	2-Hydroxyhexadecanoic acid	C_16_H_32_O_3_	45.11		271.22732	-	+	+	+	+
71 ^1^	Linoleic acid	C_18_H_32_O_2_	45.81		279.23241	+	+	+	+	+
72 ^1^	Oleic acid	C_18_H_34_O_2_	46.89		281.24806	+	+	+	+	+

^1^ Confirmed by standard. + compound present; - compound not present.

**Table 3:** Antioxidant activity (EC_50_ values, mg/mL) of extracts from *Cladium mariscus* (sawgrass) seeds: radical scavenging on DPPH•, ABTS•+, and NO radicals, ferric reducing antioxidant power (FRAP) and metal-chelating activities on copper (CCA) and iron (ICA).

Sample	DPPH•	ABTS•+	NO	FRAP	CCA	ICA
Water	5.10 ± 0.27 ^e^	1.38 ± 0.16 ^c^	8.19 ± 0.89 ^b^	2.23 ± 0.07 ^d^	2.10 ± 0.06 ^d^	2.64 ± 0.05 ^b^
Acetone	1.50 ± 0.07 ^d^	1.85 ± 0.20 ^d^	>10	0.97 ± 0.02 ^c^	2.09 ± 0.02 ^d^	>10
Aq. Acetone	0.21 ± 0.01 ^a^	0.13 ± 0.01 ^a^	>10	0.13 ± 0.00 ^a^	0.83 ± 0.01 ^b^	>10
Ethanol	0.93 ± 0.12 ^c^	0.68 ± 0.02 ^b^	>10	0.37 ± 0.01 ^b^	2.93 ± 0.06 ^e^	>10
Aq. Ethanol	0.38 ±0.02 ^ab^	0.22 ± 0.01 ^a^	>10	0.36 ± 0.01 ^b^	1.14 ± 0.04 ^c^	>10
BHA *	0.60 ± 0.03 ^bc^	0.33 ± 0.02 ^a^		0.16 ± 0.01 ^a^		
EDTA *					0.16 ± 0.00 ^a^	0.03 ± 0.01 ^a^
Ascorbic acid *			1.71 ± 0.02 ^a^			

* Positive controls. Values represent the mean ± SD of at least three experiments performed in triplicate (*n* = 9). In each column, different letters “a–e” mean significant differences (*p* < 0.05).

**Table 4:** Enzyme inhibitory activity (mg standard equiv./g extract DW) of extracts from *Cladium mariscus* (sawgrass) seeds: acetyl- (AChE) and butyryl-cholinesterase (BChE), tyrosinase, α-glucosidase, and α-amylase.

Sample	AChE (mg GALAE/g)	BChE (mg GALAE/g)	Tyrosinase (mg KAE/g)	α-Glucosidase (mmol ACAE/g)	α-Amylase (mmol ACAE/g)
Water	3.73 ± 0.13 ^b^	5.13 ± 0.78 ^ab^	61.81 ± 0.50 ^c^	1.05 ± 0.01 ^b^	1.12 ± 0.02 ^a^
Acetone	3.89 ± 0.17 ^ab^	5.05 ± 0.31 ^ab^	55.04 ± 0.69 ^d^	1.05 ± 0.01 ^b^	0.81 ± 0.01 ^c^
Aq. Acetone	3.92 ± 0.05 ^ab^	3.47 ± 0.48 ^b^	70.26 ± 1.59 ^ab^	1.10 ± 0.02 ^a^	0.82 ± 0.02 ^c^
Ethanol	4.21 ± 0.19 ^a^	n.a.	68.64 ± 0.30 ^b^	1.06 ± 0.01 ^b^	0.95 ± 0.02 ^b^
Aq. Ethanol	3.83 ± 0.17 ^ab^	6.02 ± 1.39 ^a^	70.99 ± 0.57 ^a^	1.07 ± 0.01 ^b^	0.92 ± 0.03 ^b^

Values represent the mean ± SD of at least three experiments performed in triplicate (*n* = 9). In each column, different letters “a–d” mean significant differences (*p* < 0.05). GALAE: galantamine equivalent, KAE: kojic acid equivalent, ACAE: acarbose equivalent, n.a.: not active.

**Table 5:** Anti-inflammatory activity (% NO decrease) of extracts from *Cladium mariscus* (sawgrass) seeds.

Sample	NO Decrease (%)
Water	36.45 ± 7.37 ^cd^
Acetone	26.98 ± 3.80 ^d^
Aq. Acetone	n.a.
Ethanol	38.05 ± 3.02 ^c^
Aq. Ethanol	60.09 ± 4.11 ^b^
L-NAME *	73.40 ± 4.28 ^a^

* Positive control, tested at 200 μg/mL. Values represent the mean ± SD (*n* = 6). Different letters “a–d” mean significant differences (*p* < 0.05). n.a.: not active.

## Data Availability

The dataset is available upon request from the corresponding author.

## Supplementary Materials

The following supporting information can be downloaded at: https://www.mdpi.com/article/10.3390/plants11212910/s1, Figure S1: Total ion chromatogram (positive (A) and negative (B) modes) of *C. mariscus* water extract. Figure S2: Total ion chromatogram (positive (A) and negative (B) modes) of *C. mariscus* acetone extract. Figure S3: Total ion chromatogram (positive (A) and negative (B) modes) of *C. mariscus* aqueous acetone extract. Figure S4: Total ion chromatogram (positive (A) and negative (B) modes) of *C. mariscus* ethanol extract. Figure S5: Total ion chromatogram (positive (A) and negative (B) modes) of *C. mariscus* aqueous ethanol extract.
