# Supplementary material for: Nutritional and Phyto-Therapeutic Value of the Halophyte Cladium mariscus L. (Pohl.): A Special Focus on Seeds
Source: Plants (Basel). 2022 Oct 29;11(21):2910. doi: 10.3390/plants11212910 (PMC9657221; doi:10.3390/plants11212910)
Supplement: Supplementary file 1 [file plants-11-02910-s001.zip › plants-1997679-supplementary.pdf]

Nutritional and Phyto-therapeutic Value of the Halophyte *Cladium mariscus* L. (Pohl.): a Special Focus on Seeds

M.J. Rodrigues, L. Custódio, D. Mecha, G. Zengin, Z. Cziáky, G. Sotkó, C.G. Pereira*

*cagpereira@ualg.pt

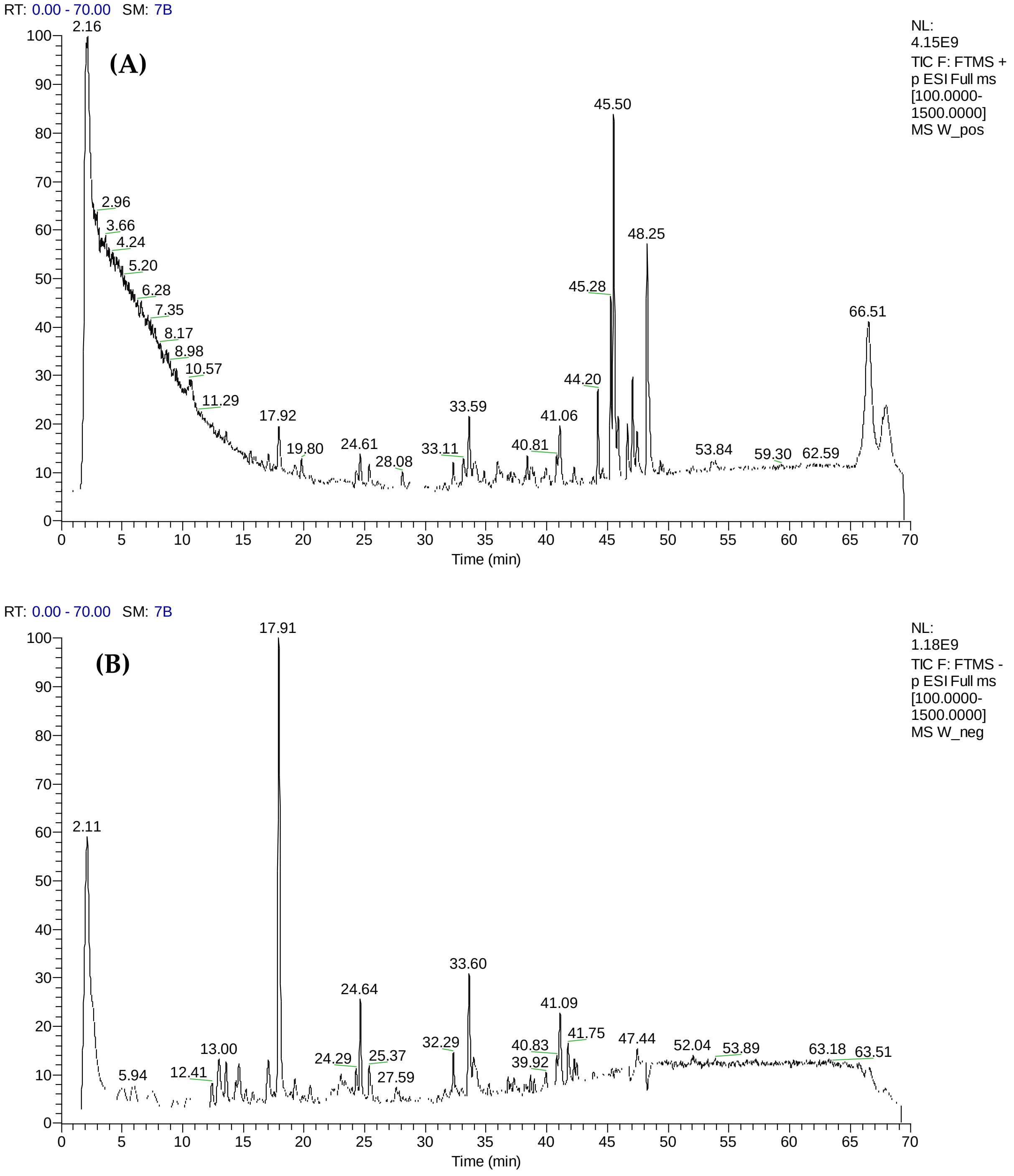

Figure S1: Total ion chromatogram (positive (A) and negative (B) modes) of *C. mariscus* water extract.

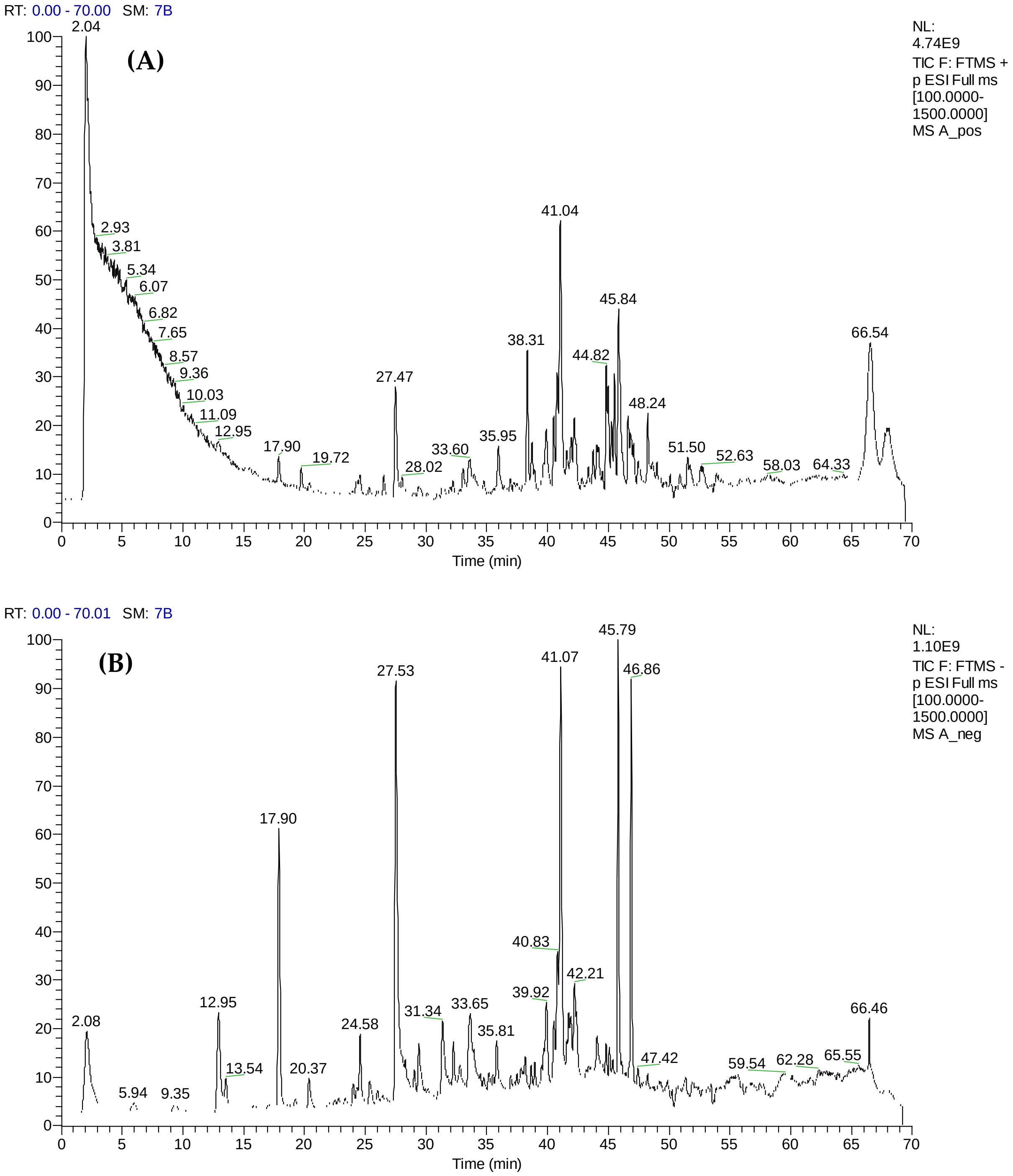

Figure S2: Total ion chromatogram (positive (A) and negative (B) modes) of *C. mariscus* acetone extract.

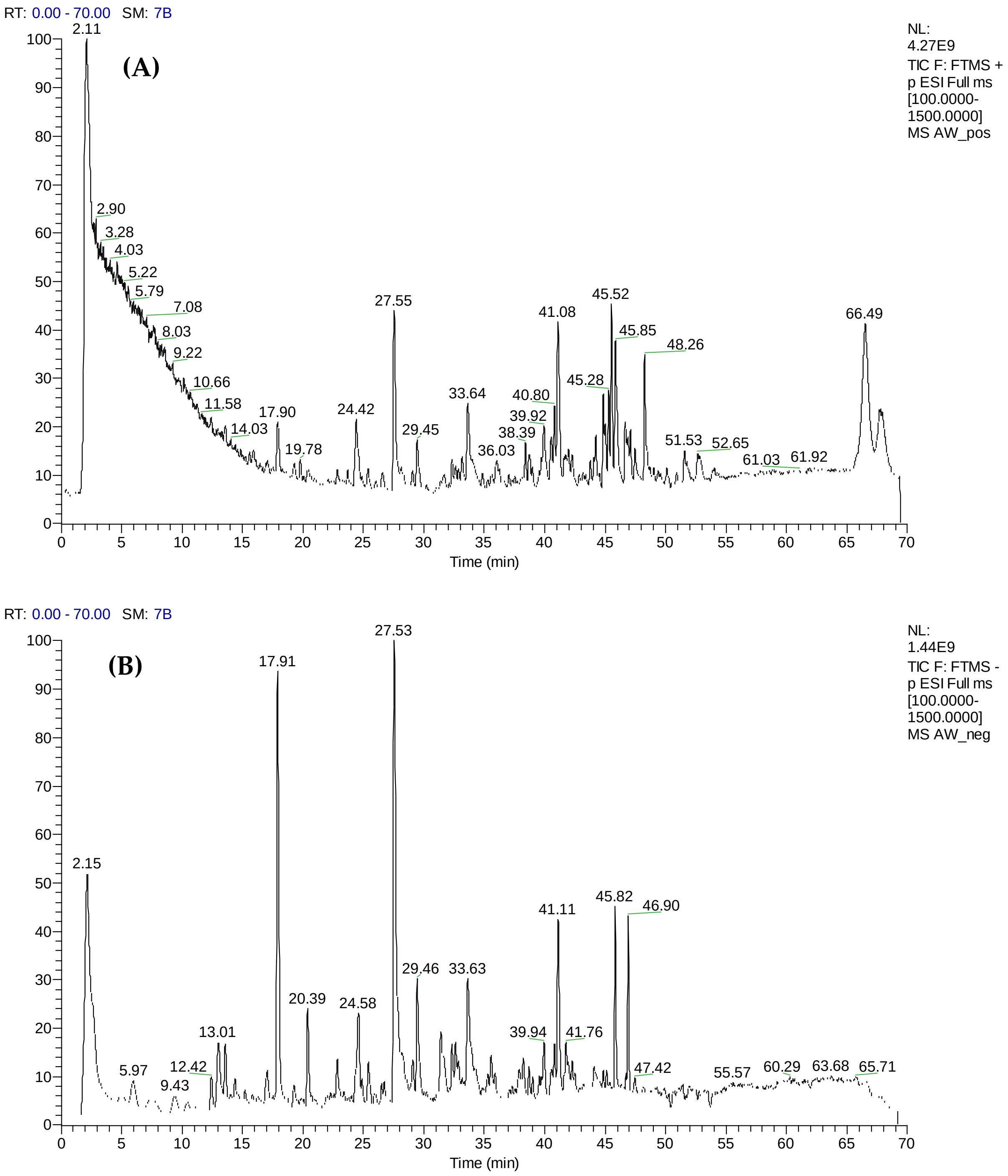

Figure S3: Total ion chromatogram (positive (A) and negative (B) modes) of *C. mariscus* aqueous acetone extract.

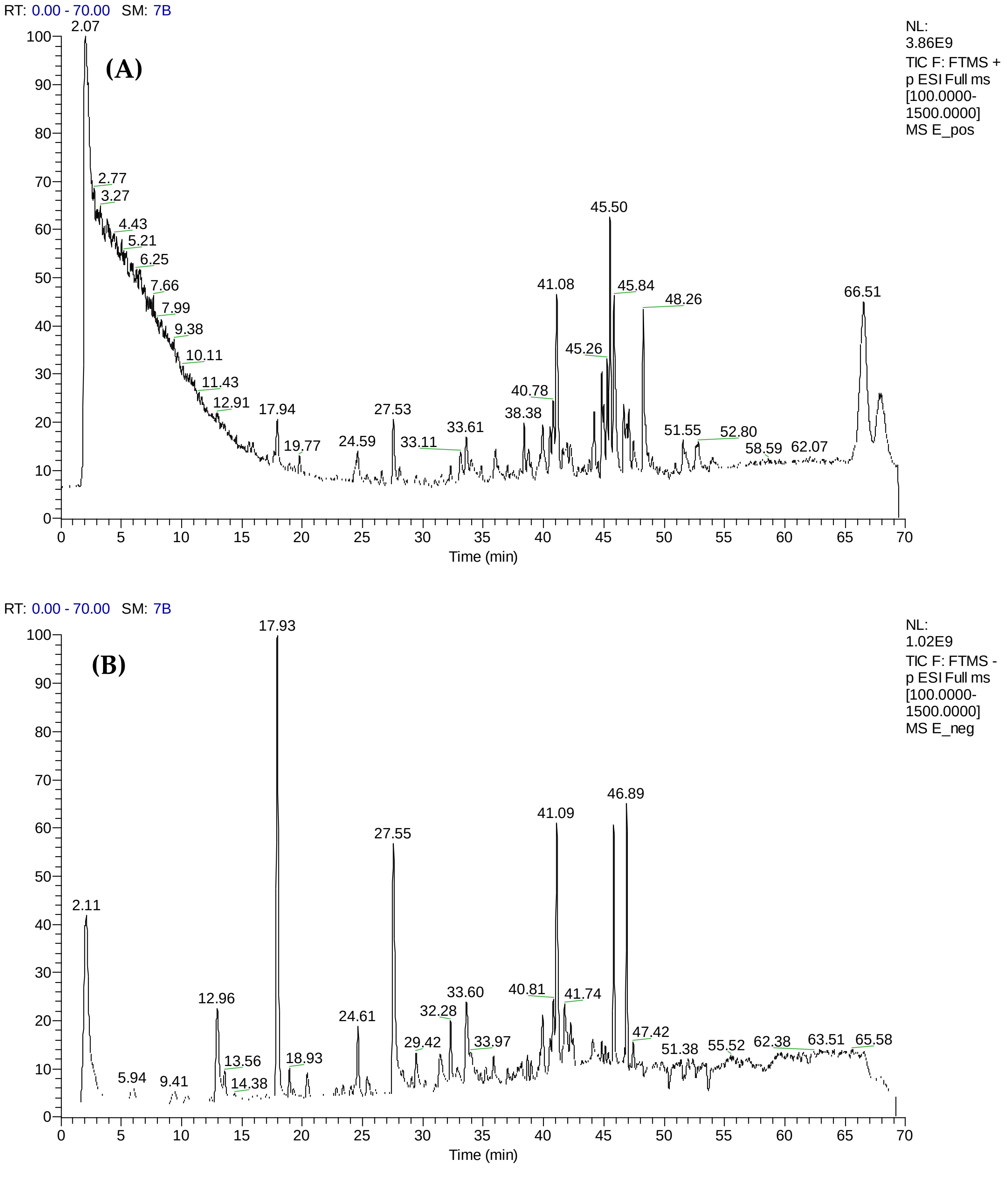

Figure S4: Total ion chromatogram (positive (A) and negative (B) modes) of *C. mariscus* ethanol extract.

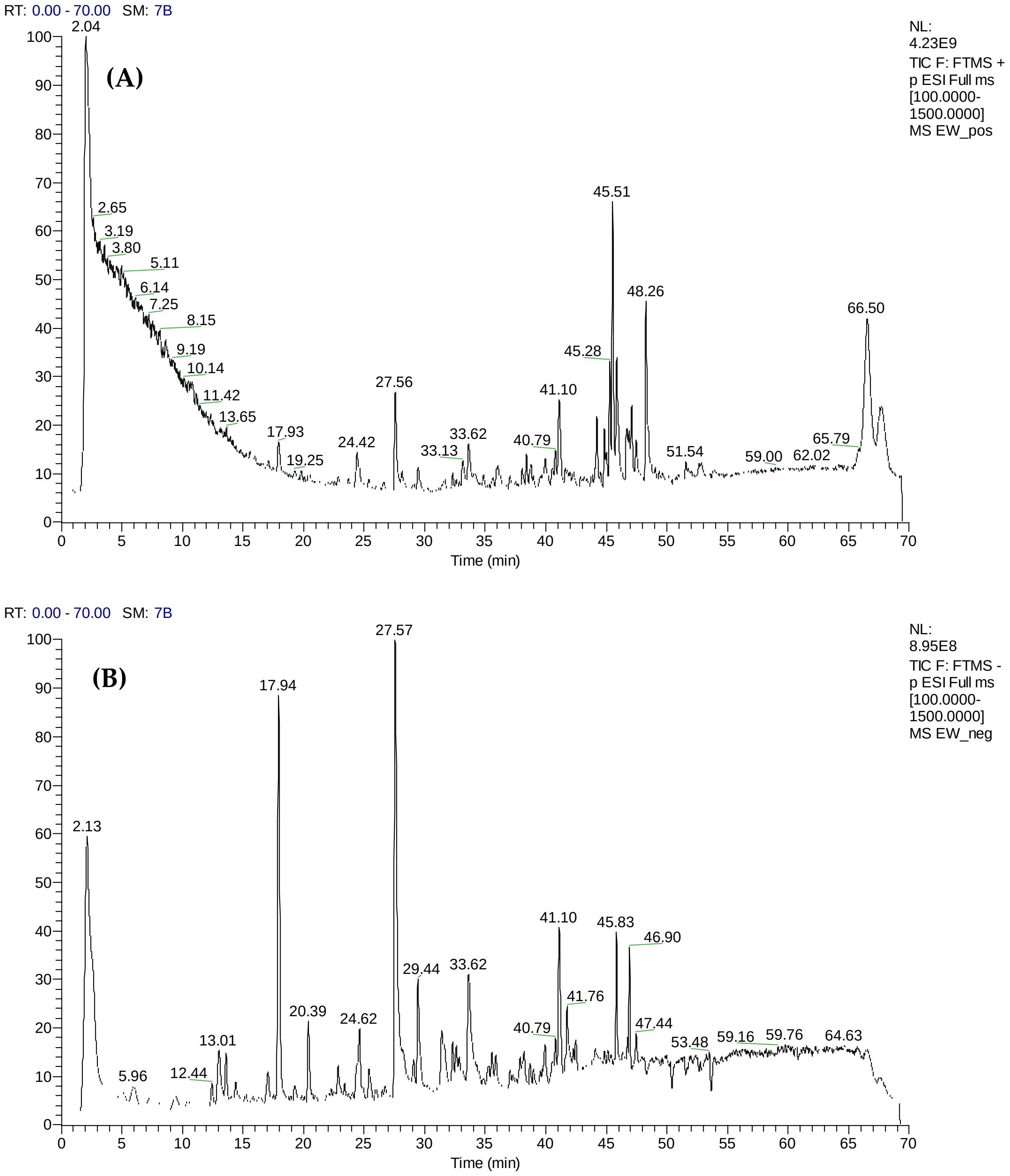

Figure S5: Total ion chromatogram (positive (A) and negative (B) modes) of *C. mariscus* aqueous ethanol extract.
